# Longitudinal alterations in motivational salience processing in ultra-high-risk subjects for psychosis

**DOI:** 10.1017/S0033291716002439

**Published:** 2016-10-04

**Authors:** A. Schmidt, M. Antoniades, P. Allen, A. Egerton, C. A. Chaddock, S. Borgwardt, P. Fusar-Poli, J. P. Roiser, O. Howes, P. McGuire

**Affiliations:** 1 Department of Psychosis Studies, King's College London, Institute of Psychiatry, Psychology and Neuroscience, London, UK; 2 Department of Psychology, University of Roehampton, London, UK; 3 Department of Psychiatry (UPK), University of Basel, Basel, Switzerland; 4 OASIS Clinic, SLaM NHS Foundation Trust, London, UK; 5 Institute of Cognitive Neuroscience, University College London, London, UK; 6 Psychiatric Imaging, MRC Clinical Sciences Centre, Hammersmith Hospital, London, UK

**Keywords:** Clinical high risk for psychosis, longitudinal studies, motivational salience, psychosis, ventral striatum

## Abstract

**Background:**

Impairments in the attribution of salience are thought to be fundamental to the development of psychotic symptoms and the onset of psychotic disorders. The aim of the present study was to explore longitudinal alterations in salience processing in ultra-high-risk subjects for psychosis.

**Method:**

A total of 23 ultra-high-risk subjects and 13 healthy controls underwent functional magnetic resonance imaging at two time points (mean interval of 17 months) while performing the Salience Attribution Test to assess neural responses to task-relevant (adaptive salience) and task-irrelevant (aberrant salience) stimulus features.

**Results:**

At presentation, high-risk subjects were less likely than controls to attribute salience to relevant features, and more likely to attribute salience to irrelevant stimulus features. These behavioural differences were no longer evident at follow-up. When attributing salience to relevant cue features, ultra-high-risk subjects showed less activation than controls in the ventral striatum at both baseline and follow-up. Within the high-risk sample, amelioration of abnormal beliefs over the follow-up period was correlated with an increase in right ventral striatum activation during the attribution of salience to relevant cue features.

**Conclusions:**

These findings confirm that salience processing is perturbed in ultra-high-risk subjects for psychosis, that this is linked to alterations in ventral striatum function, and that clinical outcomes are related to longitudinal changes in ventral striatum function during salience processing.

## Introduction

According to the aberrant salience model of psychosis (Heinz, 2002; Kapur, 2003; Howes & Murray, 2014), psychotic symptoms develop as a result of the inappropriate assignment of salience to contextually irrelevant internal and external experiences. This model is supported by evidence that patients with schizophrenia respond faster to task-irrelevant stimulus features than healthy controls (HCs) (Pankow *et al.* 2016), and that patients with prominent delusions rate irrelevant stimuli as more potentially rewarding than patients without delusions (Roiser *et al.* 2009). This ‘aberrant’ attribution of salience is also evident in people at ultra-high risk (UHR) for psychosis, who are more likely to attribute salience to irrelevant stimulus features than HCs, with this tendency again related to the severity of abnormal beliefs (Roiser *et al.* 2013).

Experiments in animals suggest that stimuli become motivationally salient when the release of dopamine in the striatum coincides with their perception (Schultz *et al.* 1997; Kapur, 2003). In healthy individuals, aberrant salience measures are positively associated with ventral striatal (VS) presynaptic dopamine levels (Boehme *et al.* 2015). Dopamine function in the striatum is abnormally elevated in both schizophrenia (Reith *et al.* 1994; Laruelle *et al.* 1996; Breier *et al.* 1997; Laruelle *et al.* 1999; Abi-Dargham *et al.* 2000; Kumakura *et al.* 2007; Howes & Kapur, 2009; Howes *et al.* 2012) and UHR subjects (Howes *et al*. 2009*b*, 2011*a*, *b*; Egerton *et al.* 2013; Mizrahi *et al.* 2014) and the aberrant salience hypothesis proposes that this causes attribution of salience to irrelevant stimuli (Heinz & Schlagenhauf, 2010; Winton-Brown *et al.* 2014). In addition, it has been hypothesized that because dopaminergic neurons may show more burst firing in psychosis (Goto & Grace, 2005; Winton-Brown *et al.* 2014) the normal phasic dopaminergic response to relevant stimuli may become relatively diminished due to the high level of noise in the system (Heinz, 2002; Kapur, 2003; Howes *et al.* 2009*a*). Psychosis may thus be associated with a reduced attribution of salience to relevant stimuli as well as increased attribution of salience to irrelevant stimuli. This is consistent with data from recent studies in UHR subjects and in patients with psychosis, which report impairments in both forms of salience processing (Roiser *et al.* 2009, 2013, Pankow *et al*. 2016).

Data from functional neuroimaging studies suggest that UHR subjects and patients with psychosis show altered activation in the VS during tasks that engage motivational salience processing. A recent meta-analysis suggested that reduced VS responses occur in patients with schizophrenia spectrum disorders relative to controls during the processing of contextually relevant information and that left VS hypoactivation was more severe in patients with high scores of negative symptoms (Radua *et al.* 2015). The relationship between VS activation during reward prediction and positive symptoms requires further investigation because only six studies were available (Simon *et al.* 2010; Esslinger *et al.* 2012; Nielsen *et al.* 2012*a*; Roiser *et al.* 2013; Wotruba *et al.* 2014; de Leeuw *et al.* 2015) and there was residual heterogeneity among them (Radua *et al.* 2015). Interestingly, individual treatment with antipsychotics was associated with a normalization of VS activation during reward prediction, and this improvement was associated with the improvement of positive symptoms (Nielsen *et al.* 2012*b*). With respect to contextually irrelevant information, it has been shown that striatal activation during incorrect distracter trials was positively correlated with aberrant salience symptoms in schizophrenia patients (Ceaser & Barch, 2015). In UHR subjects, the VS response to irrelevant stimulus features was found to be associated with the severity of abnormal beliefs (Roiser *et al.* 2013). However, it is not known if altered VS activation during salience processing normalizes in UHR individuals whose psychotic symptoms have remitted.

The Salience Attribution Test (SAT) is a paradigm that can be used to assess task-relevant and task-irrelevant motivational salience responses, termed adaptive and aberrant salience, respectively (Roiser *et al.* 2009, 2010). Our objective was to assess the relationship between changes in clinical features in a UHR cohort and longitudinal changes in VS activation elicited during the SAT paradigm. Our first hypothesis was that at clinical presentation, UHR subjects would show increased aberrant but reduced adaptive salience processing compared with HCs, and that these differences would be associated with concomitant alterations in VS activation. Our second hypothesis was that clinical improvements the UHR subjects subsequent to presentation would be associated with a longitudinal normalization of behavioural and neural responses during salience processing.

## Method

### Participants

A total of 29 individuals who met the Comprehensive Assessment of At-Risk Mental States (CAARMS) (Yung *et al.* 2005) criteria for the UHR state were recruited from Outreach and Support in South London (OASIS; Fusar-Poli *et al.* 2013*b*), a clinical service for people at high risk for psychosis. According to international standard UHR criteria (for a comprehensive review, see Fusar-Poli *et al.* 2013*a*), inclusion required the presence of one or more of the following: (i) presence of attenuated psychosis symptoms (APS); (ii) genetic risk and deterioration syndrome (GRD); or (iii) brief limited and intermittent psychotic symptoms (BLIPS). In all, 24 individuals were included based on APS, three based on BLIPS and two based on APS + GRD. Following presentation, all subjects were provided with clinical care from OASIS (Fusar-Poli *et al.* 2013*b*). Three subjects received antipsychotic medication and were thus excluded from the analysis. Of the subjects, 26 also received cognitive–behavioural therapy (CBT), which at the time of writing had been completed in nine subjects (26 sessions on average, range 14–65). Of the subjects, seven received low-dose antidepressants: five citalopram (3 × 20, 1 × 40 mg, and 1 × unknown dose), one mirtazapine (dose unknown) and one sertraline (100 mg). The specific treatments offered by OASIS have been detailed elsewhere (Fusar-Poli *et al.* 2015). All subjects were managed in the community, attending regular out-patient appointments.

A total of 15 HCs from the same geographical area were recruited via local advertisements. Absence of psychiatric illness history was confirmed with the Mini International Neuropsychiatric Inventory (Sheehan *et al.* 1998). None of the HC subjects had a history of neurological illness, or Diagnostic and Statistical Manual of Mental Disorders, 4th edition (DSM-IV) drug or alcohol dependence (American Psychiatric Association, 2013). All subjects provided informed written consent to participate and the study was approved by the local National Health Service Research Ethics Committee.

### The SAT

The SAT has been previously described in detail elsewhere (Roiser *et al.* 2009, 2010). In brief, the SAT is a speeded-response game, rewarded with money, which measures responses to cue features, which can be either task-relevant or task-irrelevant. On each trial of the task, participants were required to respond to a briefly presented square. Before the square appeared, a cue was shown indicating the likelihood of obtaining a reward for the forthcoming response. Participants received a monetary reward on 50% of trials, with more money awarded for faster responses. The cues varied in two different visual dimensions; colour (red or blue) and shape (animals or household objects). One of these cue dimensions was task-relevant and the other task-irrelevant. One task-relevant feature was highly associated with receiving a reward, with 87.5% of these trial types rewarded (e.g. blue stimuli). The other task-relevant dimension (e.g. red stimuli) was not rewarded on any trials. For the task-irrelevant dimension, an equal proportion of both features (e.g. animal and household stimuli) was rewarded. Participants were not informed about these contingencies, which remained the same over the two blocks of 64 trials within a testing session, and instead had to learn them over successive trials of the task. To avoid practice effects between baseline and follow-up, four different versions of the task were used, counterbalanced across participants, each with a different stimulus feature (blue, red, animal or household) rewarded with high probability.

Participants performed the task on two occasions, while being scanned using functional magnetic resonance imaging (fMRI). The baseline assessment was performed at the time of clinical presentation. The follow-up assessment was carried out approximately 17 months later. On each visit they performed the same version of the task twice. The SAT provides behavioural measures of adaptive (relevant) and aberrant (irrelevant) motivational salience on the basis of reaction times (RTs: implicit salience) and visual analogue scale (VAS) ratings from 0 to 100% (explicit salience). Implicit adaptive salience is defined as the speeding of responses on high- relative to low-probability reward trials. Explicit adaptive salience is defined as the increase in VAS ratings on high- relative to low-probability reward trials. Implicit aberrant salience and explicit aberrant salience are defined as the absolute difference in RT and VAS ratings, respectively, between the two levels of the task-irrelevant stimulus dimension (Roiser *et al.* 2009).

### Behavioural analysis

Behavioural scores on the SAT were analysed using a repeated-measures analysis of variance (ANOVA) with time as within-subject and group as between-subject factors and years of education as a covariate. To test for group differences at baseline and follow-up separately, univariate ANOVA with education as covariate was used. Using box-and-whisker plots on each SAT measure for both groups separately, two HCs and two UHR subjects were excluded as outliers.

### fMRI data acquisition and analysis

Scanning was performed on a whole-body 3 T MRI General Electric (USA) system. During each of the four scanning runs (two per day), we acquired T2*-weighted echo-planar images (EPIs) with the following parameters: 50 axial slices (sequential and top-down acquisition) of 2.4 mm thickness, 2.7 mm interslice gap, field of view 240 mm^2^ and matrix size 64 × 64. The repetition time was 2.5 s and the echo time 25 ms. A total of 237 image volumes were acquired in a single functional run.

EPIs were analysed using an event-related design with SPM12 (www.fil.ion.ucl.ac.uk/spm). Pre-processing was performed for each subject and time point separately. In brief, slice-timing correction was first performed on each volume using the middle slice as the reference. The images were then realigned to the first image in the series (following removal of dummy scans), spatially normalized to the Montreal Neurological Institute (MNI) template and smoothed with a Gaussian kernel of 8 mm full half-width maximum (FWHM). All images underwent visual inspection and participants with a high number of severely corrupted images and/or gross artefacts were excluded (two HCs and one UHR). Additionally, all images were checked for movement artefacts, and all scans with more than 5 mm deviation from the previous scan in any dimension, resulting in corrupted volumes, were excluded and replaced with the average of the neighbouring volumes (5.1% in HCs and 1.5% in UHRs). Subjects with more than 10% corrupted volumes were excluded (two UHRs). In the final sample of 23 UHR subjects, 19 subjects were included based on BLIPS, three based on BLIPS and one based on APS + GRD.

Voxel-wise maximum likelihood parameter estimates were calculated during the first-level analysis using the general linear model. Our design matrix included an autoregressive AR(1) model of serial correlations and a high-pass filter with a cut-off of 128 s. The onsets of each event were convolved with the SPM synthetic haemodynamic response function. In this model, we included four ‘cue’ regressors, representing the different cue types and an ‘outcome’ regressor representing the time points when reward feedback was provided during the task. Cues on which participants failed to respond entirely were excluded from the analysis (regressor of no interest) due to the possibility that participants were not attending during the trial. Eight contrast images were generated per participant: adaptive and aberrant reward prediction at baseline and follow-up separately; average images over both visits for adaptive and aberrant reward prediction (to test for main effect of group); and two images subtracting the contrast vector of baseline from follow-up (to test for the main effect of time and time x group interaction). Adaptive reward prediction contrasts were defined as: high-probability reward cue features minus low-probability reward cue features across the task-relevant dimension. Aberrant reward prediction contrasts were defined as subjective ‘high-probability’ reward cue features minus subjective ‘low-probability’ reward cue features (based on the subject's VAS ratings for that run) across the task-irrelevant dimension (Roiser *et al.* 2010).

Two-sample tests were conducted at the second level to test for group effects at baseline and follow-up separately, as well as to test for main effects of group and time and for time x group interactions. Significance was assessed at a cluster-level threshold of *p* < 0.05 family-wise error (FWE) corrected across the whole brain, using an uncorrected cluster-forming threshold of *p* < 0.001 (Petersson *et al.* 1999; Woo *et al.* 2014) with an extent threshold of 20 voxels. We also focused our analysis on the VS as this was part of our primary hypothesis, using a voxel-level approach. The VS region of interest was defined using coordinates taken from a previous SAT fMRI study in an independent UHR cohort (Roiser *et al.* 2013): right (*x* = 12; *y* = 12; *z* = −3) and left (*x* = −12; *y* = 9; *z* = −3). Small volume correction was applied for this analysis using 15 mm spheres around these coordinates (Roiser *et al.* 2013) and a voxel-level threshold of *p* < 0.05 FWE corrected was considered significant. As groups differed in years of education, this variable was added as a covariate in the second-level model.

### Relationships between brain activation, behaviour and symptoms

Relationships between neural responses and behavioural and clinical features were identified by including outcome measures from the SAT, CAARMS and Global Assessment of Functioning (GAF) as covariates in second-level models. The same procedure for correction for multiple comparisons as described above was employed. Relationships between behavioural salience responses and symptomatology in UHR subjects were tested with Pearson's correlation coefficients using the Statistical Package for the Social Sciences (SPSS 16, SPSS Inc., USA).

## Results

### Demographical and clinical features

The two groups did not differ in age, gender, handedness, intelligence quotient or cigarette, alcohol, cannabis and cocaine consumption, but HCs had more years of education (therefore, all group comparisons were covaried for years of education). At baseline, UHR subjects had higher scores on CAARMS positive and negative symptoms and lower scores on the GAF. Over time, the UHR group showed significant improvements in CAARMS positive and negative symptoms, but not in GAF scores (Table 1).

**Table 1.** Demographical and clinical characteristics of the study sample

	Healthy controls (*n* = 13)	Ultra-high-risk subjects (*n* = 23)	Group statistics
At baseline			
Mean age, years (s.d.) [range]	24.38 (5.32) [20–36]	21.57 (3.55) [18–29]	*t*_34_ = 1.709, *p* = 0.104^a^
Female/male, *n*	3/10	11/12	χ^2^ = 2.141, *p* = 0.143^a^
Handedness, right/left, *n*	11/2	22/1	χ^2^ = 1.324, *p* = 0.250^a^
Mean duration of education, years (s.d.)	14.77 (1.64)	12.52 (2.25)	*t*_34_ = 3.436, *p* = 0.002^a^
Mean NART FSIQ (s.d.)	111.50 (6.10)	107.04 (9.07)	*t*_34_ = 1.578, *p* = 0.124^a^
Mean number of cigarettes, per day (s.d.)	2.69 (4.38)	5.57 (8.13)	*t*_34_ = 1.376, *p* = 0.178^a^
Mean number of alcohol units, per week (s.d.)	11 (10.52)	6.87 (9.28)	*t*_34_ = 1.179, *p* = 0.251^a^
Cannabis consumption, yes/no, *n*	8/5	14/9	χ^2^ = 0.002, *p* = 0.0968^a^
Cocaine consumption, yes/no, *n*	4/9	8/15	χ^2^ = 0.060, *p* = 0.806^a^
Mean GAF (s.d.)	84.15 (4.88)	59.74 (7.61)	*t*_34_ = 11.706, *p* < 0.001^a^
Mean CAARMS positive symptoms (s.d.)^c^	0.54 (1.20)	7.65 (3.81)	*t*_34_ = −8.262, *p* < 0.001^a^
Mean CAARMS negative symptoms (s.d.)^c^	0.17 (0.58)	6.39 (3.29)	*t*_34_ = −7.025, *p* < 0.001^a^
At follow-up			
Mean age, years (s.d.) [range]	25.70 (5.33) [21–37]	22.96 (3.48) [19–30]	*t*_34_ = 1.668, *p* = 0.113^a^
Mean number of cigarettes, per day (s.d.)	–	3.13 (5.65)	*t*_22_ = 1.611, *p* = 0.121^b^
Mean number of alcohol units, per week (s.d.)	–	7.74 (10.82)	*t*_22_ = 0.506, *p* = 0.618^b^
Cannabis consumption, yes/no, *n*	–	14/9	χ^2^ = 0.000, *p* = 1^b^
Cocaine consumption, yes/no, *n*	–	8/15	χ^2^ = 0.000, *p* = 1^b^
Mean GAF (s.d.)	–	62.39 (15.78)	*t*_22_ = 0.896, *p* = 0.38^b^
Mean CAARMS positive symptoms (s.d.)^c^	–	5.22 (4.88)	*t*_22_ = 2.811, *p* = 0.010^b^
Mean CAARMS negative symptoms (s.d.)^c^	–	4.22 (4.35)	*t*_17_ = 2.663, *p* = 0.016^b^

s.d., Standard deviation; NART FSIQ, National Adult Reading Test full-scale intelligence quotient; GAF, Global Assessment of Functioning; CAARMS, Comprehensive Assessment of At-Risk Mental States.

a Two-sample *t* tests and χ^2^ tests between groups, respectively.

b Paired tests and χ^2^ tests within ultra-high-risk subjects between baseline and follow-up assessment.

c CAARMS positive symptoms were the sum of severity scores for unusual thought content (abnormal belief), non-bizarre ideas, perceptual abnormalities and disorganized speed; negative symptoms were the sum of severity scores for alogia, avolition/apathy and anhedonia.

### Behavioural data

#### Aberrant attribution of salience

Across both visits, UHR subjects showed significantly higher implicit aberrant salience than HC subjects (*F*_1,34_ = 6.718, *p* = 0.014), and there was a trend for a group × time interaction (*F*_1,34_ = 3.225, *p* = 0.081). There was also a trend for a group × time interaction for explicit aberrant salience (*F*_1,34_ = 3.325, *p* = 0.077). Based on our *a priori* hypotheses we constructed linear contrasts at each time point to test for the predicted group differences in aberrant salience.

**Fig. 1.**
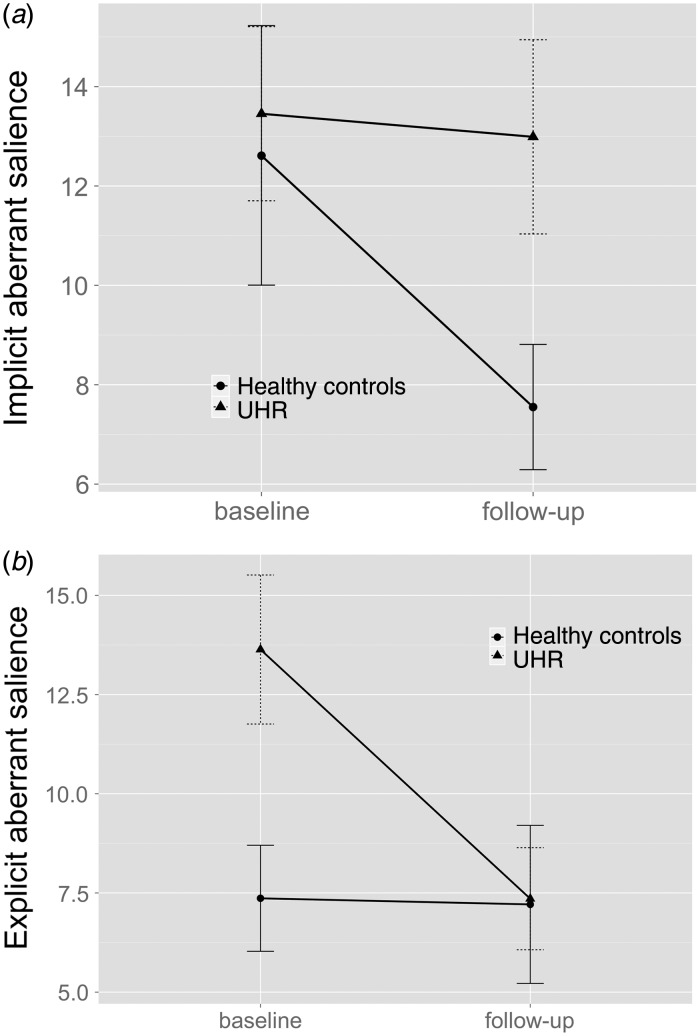
(*a*) Implicit (reaction times, ms) and (*b*) explicit (visual analogue scale) scores for aberrant motivational salience processing in healthy controls and subjects at ultra-high risk (UHR) for psychosis. Values are means, with standard errors represented by vertical bars.

At baseline, UHR subjects were more likely than HCs to attribute salience to irrelevant cue features (explicit aberrant salience) (*F*_1,34_ = 4.732, *p* = 0.037), but did not exhibit greater implicit aberrant salience than HCs (*F*_1,34_ = 0.964, *p* = 0.333). At follow-up the group difference in explicit aberrant salience was no longer significant (*F*_1,34_ = 0.061, *p* = 0.806), but HCs had significantly lower implicit aberrant scores than the UHR group (*F*_1,34_ = 12.296, *p* = 0.001) due to a reduction in this measure over time (Figs. 1*a* and *b*).

Within the UHR group we detected no significant correlations between aberrant salience responses and psychotic symptoms (baseline, follow-up, change over time).

#### Adaptive attribution of salience

Across both visits, the UHR group had lower implicit adaptive salience scores than HCs (*F*_1,34_ = 11.472, *p* = 0.002), as well as lower explicit adaptive salience scores (*F*_1,34_ = 5.493, *p* = 0.035). There was also a significant group x time interaction for explicit adaptive salience (*F*_1,34_ = 4.157, *p* = 0.049).

**Fig. 2.**
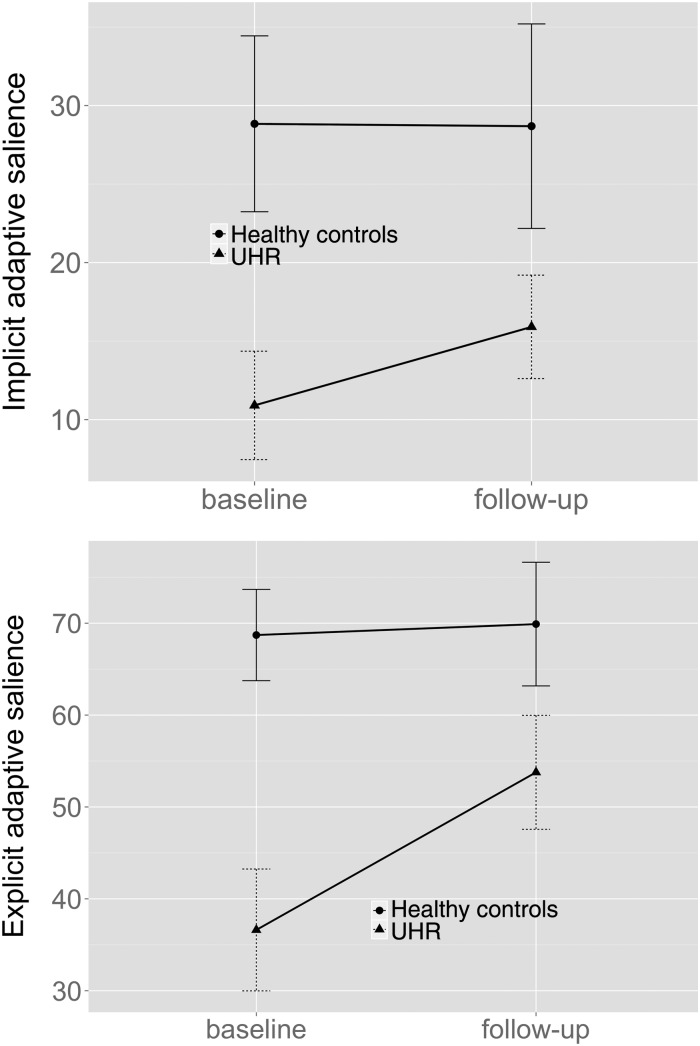
(*a*) Implicit (reaction times, ms) and (*b*) explicit (visual analogue scale) scores for adaptive motivational salience processing in healthy controls and subjects at ultra-high risk (UHR) for psychosis. Values are means, with standard errors represented by vertical bars.

At baseline, UHR subjects had significantly lower implicit adaptive salience than HCs (*F*_1,34_ = 13.866, *p* = 0.001) and also exhibited significantly lower explicit adaptive salience (*F*_1,34_ = 9.043, *p* = 0.005). Both of these group differences were no longer significant at follow-up (implicit adaptive salience: *F*_1,34_ = 3.733, *p* = 0.062; explicit adaptive salience: *F*_1,34_ = 1.360, *p* = 0.252), due to improved scores in the UHR group together with relatively stable performance in HCs (Figs. 2*a* and *b*).

Within the UHR group, explicit adaptive salience scores at follow-up were negatively correlated with the severity of abnormal beliefs (*r* = −0.674, *p* < 0.001) (online Supplementary Fig. S1A) and of positive symptoms (*r* = −0.653, *p* < 0.001) (online Supplementary Fig. S1B), and positively correlated with the level of global functioning (*r* = 0.497, *p* = 0.014) (online Supplementary Fig. S1C).

All behavioural results remained after excluding the UHR subject with a later transition to psychosis (online Supplementary information 2A).

### Activation during salience processing

#### Aberrant reward prediction

There were no significant effects of group or time, and no group x time interactions. There were also no significant group differences in responses to irrelevant cues at either baseline or follow-up.

#### Adaptive reward prediction

**Fig. 3.**
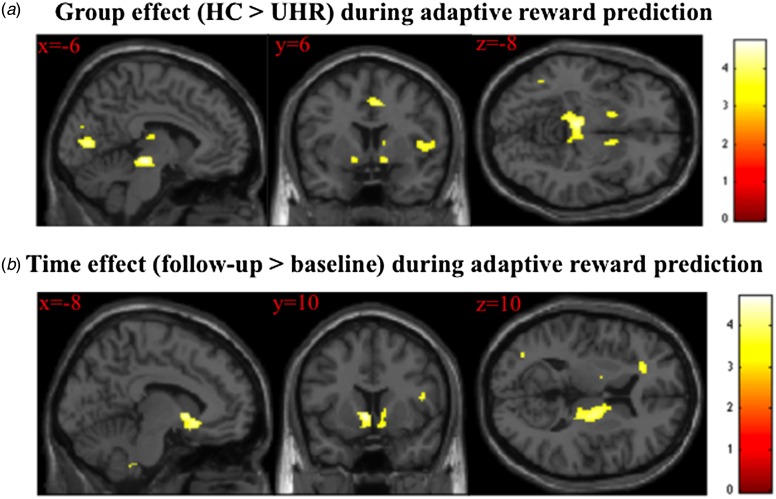
(*a*) Greater activation during adaptive reward prediction in healthy controls (HC) compared with ultra-high-risk (UHR) subjects across both visits. (*b*) Greater activation at follow-up relative to baseline during adaptive reward prediction across both groups. Images are displayed at a cluster-forming threshold of *p* < 0.001 uncorrected, with an extent threshold of 20 voxels. Colour bars indicate *t* values.

Across both time points, UHR subjects showed less activation than HCs in the VS, calcarine sulcus and midbrain bilaterally and in the left cuneus and middle temporal gyrus (main effect of group: Fig. 3*a*, online Supplementary Table S1). Across both groups, activation during adaptive reward prediction was greater at follow-up than at baseline in the bilateral VS and right thalamus (main effect of time: Fig. 3*b*, online Supplementary Table S2). No significant group × time interactions were found for adaptive reward prediction.

At baseline, the UHR group showed significantly less activation than HCs in the VS bilaterally and the left parahippocampal and middle temporal gyrus, and cerebellum during adaptive reward prediction (online Supplementary Table S3). At follow-up, the UHR group continued to show significantly less activation in the VS bilaterally (online Supplementary Table S4). All results remained after excluding the UHR subject with a later transition to psychosis (online Supplementary information S2B).

There were no significant relationships between neural responses from the aberrant and adaptive reward prediction contrast and behavioural scores on the SAT (baseline, follow-up, change over time).

### Relationship between longitudinal changes in clinical features and brain activation

#### Aberrant salience

There were no significant relationships between changes in clinical features and longitudinal changes in brain activation during aberrant reward prediction.

#### Adaptive salience

**Fig. 4.**
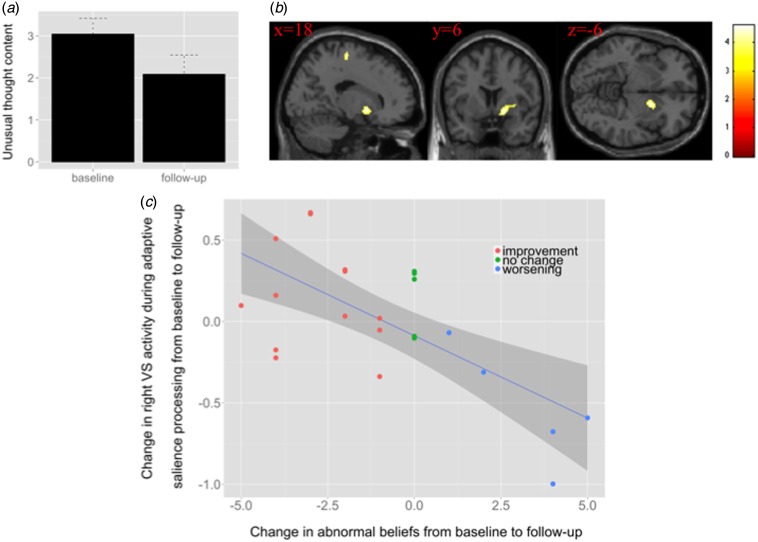
(*a*) Unusual thought content (abnormal beliefs) at baseline (mean: 3.04) and follow-up (mean: 2.09) in ultra-high-risk (UHR) subjects (*t*_22_ = 1.775, *p* = 0.09). (*b*) Negative correlation between changes in brain activation during adaptive reward prediction and changes in abnormal beliefs from baseline to follow-up in UHR subjects. The image is displayed at a cluster-forming threshold of *p* < 0.001 uncorrected, with an extent threshold of 20 voxels. The colour bar indicates *t* values. (*c*) Scatterplot of negative relationship between change in right ventral striatum (VS) activation during adaptive salience processing, taken from the peak voxel in (*b*) and change in abnormal beliefs [Comprehensive Assessment of At-Risk Mental States (CAARMS) item unusual thought content] from baseline to follow-up in UHR subjects (*r* = −0.702).

In the UHR group, there was a trend (*t*_22_ = 1.775, *p* = 0.09) for the mean severity of abnormal beliefs to improve between presentation and follow-up (Fig. 4*a*). The degree of improvement in abnormal beliefs over time was associated with the longitudinal increase in activation during adaptive reward prediction in the right VS and in the supplementary motor cortex bilaterally (Figs. 4*b* and *c*, online Supplementary Table S5). This relationship remained after excluding the UHR subject with a later transition to psychosis (online Supplementary information S2B).

There were no significant correlations between longitudinal changes in negative symptoms and in neural responses during motivational salience processing.

## Discussion

To our knowledge, this is the first longitudinal investigation of salience processing in subjects with psychotic symptoms. We explored the relationship between changes in the clinical features of people at UHR for psychosis after they had presented to clinical services and longitudinal changes in their behavioural and neural responses during aberrant and adaptive salience processing.

### Aberrant salience

Consistent with the aberrant salience model (Heinz, 2002; Kapur, 2003; Howes & Kapur, 2009), we found that UHR subjects were more likely to attribute salience to irrelevant stimuli than HCs at clinical presentation. These data are consistent with a previous report of increased explicit aberrant salience in an independent UHR sample (Roiser *et al.* 2013). A study using the SAT in first-episode schizophrenia did not find a difference in the patient sample overall, but found that aberrant salience was related to the severity of delusions and negative symptoms within the patient group (Roiser *et al.* 2009). However, it should be noted that another study found no significant differences between UHR subjects, first-episode patients and controls in aberrant salience attribution (Smieskova *et al.* 2015).

During the 17-month follow-up period, there was a reduction in explicit aberrant salience in UHR subjects, such that there was no longer a significant group difference relative to HCs. On the basis that abnormal salience processing is proposed to underlie the generation of psychotic symptoms (Roiser *et al.* 2009, 2013), we tested whether longitudinal changes in aberrant salience processing were related to changes in clinical features in the UHR subjects during the follow-up period. Although UHR subjects showed improvements clinically, there were no significant correlations between changes in these variables and longitudinal changes in behavioural measures of aberrant salience processing. It has been proposed that the link between aberrant salience and symptoms is moderated by cognitive biases (Howes & Murray, 2014), which may account for the lack of direct relationship between aberrant salience and symptom change in our data.

### Adaptive salience

The aberrant salience model proposed that adaptive salience is intact in patients with psychosis, but may become impaired as a result of treatment with antipsychotic medication (Heinz, 2002; Kapur, 2003). The first experimental study of salience processing in first-episode psychosis using the SAT found that patients showed impaired adaptive salience, and this was attributed to be an effect of antipsychotic treatment (Roiser *et al.* 2009). However, a subsequent study of largely medication-naive UHR subjects also found a trend for reduced implicit adaptive salience (Roiser *et al.* 2013). In the present study, which involved a larger patient sample, at presentation, UHR subjects showed significantly reduced adaptive salience responses. As all of our UHR subjects were naive to antipsychotic medication at this stage, these data not only suggest that adaptive salience is impaired in UHR subjects, but that this is not secondary to antipsychotic treatment. Consistent with this interpretation, a recent study found that adaptive salience processing was numerically (though not significantly) impaired in first-episode psychosis patients, but this impairment was if anything less marked in antipsychotic-treated than untreated patients (Smieskova *et al.* 2015).

Although significant behavioural differences in adaptive salience processing were only present at baseline, group differences in activation during adaptive salience processing were seen at both presentation and follow-up time points. At both time points, UHR subjects showed reduced activation relative to HCs in the VS. This is consistent with a recent meta-analysis demonstrating reduced VS activity in response to reward-predicting cues in schizophrenia spectrum disorders (Radua *et al.* 2015), and reports of altered VS activation in patients with psychosis during reward prediction error tasks (Murray *et al.* 2008; Gradin *et al.* 2011). Furthermore, within the UHR group, improvement in abnormal beliefs over the follow-up period was correlated with the degree to which VS activation increased over time during adaptive reward prediction. This finding is in line with data from unmedicated first-episode patients demonstrating a negative correlation between the severity of delusional symptoms and reward prediction signals in the VS (Esslinger *et al.* 2012). Taken with longitudinal positron emission tomography imaging findings that changes in dopamine synthesis capacity in the dorsal (associative) striatum are associated with change in clinical state (Howes *et al.* 2011*a*), our findings suggest that alterations in both the VS and dorsal striatum are linked to symptom change. A possible mechanism could be that hyperactive inputs from the hippocampus to the VS in psychosis may have an impact on dopaminergic neurons that project to more dorsal (associative) striatal areas and thereby affect dorsal striatum-related salience processing (Haber, 2003; Lodge & Grace, 2011, 2012; Modinos *et al.* 2015).

The amelioration of abnormal beliefs in UHR subjects was also associated with longitudinal increases in activation in the supplementary motor cortex to reward predicting cue features. The latter finding was not predicted, as the supplementary motor cortex is not specifically implicated in motivational salience processing. However, the SAT is a complex task that also involves sustained attention, maintaining stimulus information in memory, decision-making and response selection (Roiser *et al.* 2013), and the UHR state is associated with a broad range of cognitive impairments (Fusar-Poli *et al.* 2012). We therefore speculate that this finding in the supplementary motor cortex may be related to alterations in one or more of these processes, possibly secondary to changes in striatal function. Furthermore, UHR subjects also showed reduced activation in the calcarine sulcus, cuneus, midbrain and middle temporal gyrus across both visits during the attribution of salience to relevant stimuli, as well as reduced activation in the parahippocampal gyrus, cerebellum, midbrain, middle temporal gyrus, middle and anterior cingulate cortex, inferior frontal gyrus and insula at baseline and/or follow-up (see online Supplementary information S1 and S2B for more details). Together with the striatum, integration of these regions is important to sustain emotion and cognition, especially during the detection and processing of salient information (Seeley *et al.* 2007; Menon & Uddin, 2010). Dysfunction of this network and abnormal network switching when dealing with a relevant task at hand has been proposed to underlie the formation of psychotic symptoms (Palaniyappan & Liddle, 2012; Palaniyappan *et al.* 2013; Schmidt *et al.* 2016).

Some limitations of our study merit comment. The sample sizes were modest, largely because inclusion required that participants completed multi-modal neuroimaging assessments at both baseline and follow-up. The modest group sizes may thus have accounted for the absence of significant group differences in activation during aberrant salience processing. A further consideration is that at the time of writing, only one UHR subject had developed a psychotic disorder (all results remained after excluding this subject; see Supplementary information S2A and B for details), precluding any examination of the relationship between abnormal salience processing and the risk of transition to psychosis. In this regard, it is possible that the low conversion rate in our UHR sample may explain the lack of alterations in brain activation during aberrant salience processing. Future large-scale studies with a meaningful ratio between converters and non-converters are required to test if functional brain alterations during aberrant reward prediction are evident in UHR subjects who later develop psychosis or if the risk of transition to psychosis is more related to impaired activation when dealing with a relevant task at hand (i.e. adaptive reward prediction). Furthermore, in accordance with the aberrant salience model (Kapur, 2003), the SAT has been designed to measure abnormal motivational (reward) salience processing in psychosis and its relation to dopamine dysregulation in the VS. However, motivation is not the only form of salience (Winton-Brown *et al.* 2014), and it would be important to test ventral and dorsal (associative) striatal activation in psychosis during other forms of salience processing that are not measured using speeded response tasks. Finally, subsequent to presentation, some of the UHR subjects received CBT or low doses of antidepressants, which may have influenced our findings. In this study, the numbers of subjects receiving different forms of treatment were too small to allow for meaningful subgroup analyses and this issue would be better addressed in longitudinal studies that were explicitly linked to a clinical trial of an intervention that might be expected to improve motivational salience processing.

In summary, this study shows that UHR subjects exhibit behavioural deficits in both adaptive and aberrant salience processing at clinical presentation, which disappeared along with the remission of APS over the follow-up period. Our results further indicate VS hypoactivation in UHR subjects during adaptive reward prediction at baseline and follow-up and that the amelioration of abnormal beliefs over the follow-up period is linked to a longitudinal increase in VS activation during adaptive reward prediction.

## Supplementary material

For supplementary material accompanying this paper visit http://dx.doi.org/10.1017/S0033291716002439. click here to view supplementary material
