# Supplementary material for: Longitudinal alterations in motivational salience processing in ultra-high-risk subjects for psychosis
Source: Psychol Med. 2016 Oct 4;47(2):243–54. doi: 10.1017/S0033291716002439 (PMC5216461; doi:10.1017/S0033291716002439)
Supplement: Supplementary file 1 [file S0033291716002439sup001.doc]

**Supplementary information S1. Results in healthy controls (n=13) and all subjects at ultra high-risk for psychosis (n=23).**

***Supplementary Table S1.*** *Group effect across both visits during adaptive reward prediction*

| **Healthy controls > Ultra high-risk subjects** | | | | | |
| --- | --- | --- | --- | --- | --- |
| **Region** | **P value** | **Cluster size** | **MNI co-ordinates (X/Y/Z)** | **R/L** | **Z value** |
| Ventral striatum***** | 0.029^a^ | /† | 12/4/-10 | R | 3.48 |
| Ventral striatum***** | 0.038^a^ | /† | -14/8/-8 | L | 3.38 |
| Calcarine sulcus | 0.049^b^ | 322† | 12/-74/14 | L | 4.11 |
| Cuneus |  |  | -2/-84/20 | L | 3.40 |
| Calcarine sulcus |  |  | 22/-70/6 | R | 3.38 |
| Midbrain | 0.048^b^ | 311† | -6/-24/-10 | L | 4.10 |
| Midbrain |  |  | 6/-22/-10 | R | 3.61 |
| Middle temporal gyrus | 0.047^b^ | 328† | -48/-54/-4 | L | 3.77 |
| Superior temporal gyrus | 0.7226^b^ | 53 | -46/12/-16 | L | 3.95 |
| Cuneus | 0.1292^b^ | 223 | -14/-74/24 | L | 3.92 |
| Occipital gyrus |  |  | -26/-76/20 | L | 3.58 |
| Calcarine sulcus | 0.2111^b^ | 175 | -8/-78/6 | L | 3.77 |
| Superior patietal gyrus | 0.4378^b^ | 105 | -14/-70/44 | L | 3.77 |
| Inferior frontal gyrus | 0.2600^b^ | 155 | 52/10/2 | R | 3.68 |
| Insula |  |  | 44/8/6 | R | 3.65 |
| Cuneus | 0.2546^b^ | 157 | 18/-96/8 | R | 3.65 |
| Calcarine sulcus |  |  | 24/-86/6 | R | 3.56 |
| Supramarginal gyrus | 0.7971^b^ | 41 | 54/-36/34 | R | 3.60 |
| Middle cingulate cortex | 0.5099^b^ | 90 | 6/6/44 | R | 3.54 |
| Inferior parietal gyrus | 0.5334^b^ | 35 | -30/-44/36 | L | 3.52 |
| Precentral gyrus | 0.6493^b^ | 65 | -50/2/36 | L | 3.51 |
| Ventral striatum | 0.5412^b^ | 84 | 12/0/8 | R | 3.43 |
| Thalamus |  |  | 12/-14/8 | R | 3.37 |
| Insula | 0.8032^b^ | 40 | -36/-4/12 | L | 3.41 |
| Postcentral gyrus | 0.7103^b^ | 55 | -20/-34/54 | L | 3.40 |
| Paracentral gyrus |  |  | -14/-26/54 | L | 3.31 |
| Ventral striatum | 0.8513^b^ | 32 | -14/8/-8 | L | 3.38 |
| Thalamus | 0.8798^b^ | 27 | -4/-20/12 | L | 3.34 |

Results are reported using a cluster-forming threshold p<0.001 uncorrected, with an extent threshold of 20 voxels. **^*^**Small volume corrected. ^a^peak-level FWE-corrected, ^b^cluster-level FWE-corrected. † survives FWE correction for multiple comparisons at the cluster or voxel level.

No significant effects were found for ultra high-risk subjects > healthy controls.

**Supplementary Table S2.** *Time effect across both groups during adaptive reward prediction*

| **Follow-up activity > baseline activity** | | | | | |
| --- | --- | --- | --- | --- | --- |
| **Region** | **P value** | **Cluster size** | **MNI co-ordinates (X/Y/Z)** | **R/L** | **Z value** |
| Ventral part of head of caudate nucleus* | 0.0043^a^ | /† | -6/10/-2 | L | 4.09 |
| Ventral part of head of caudate nucleus | 0.0911^b^ | 251 | -6/10/-2 | L | 4.09 |
| Cerebellum | 0.8115^b^ | 39 | -4/-36/-46 | L | 3.84 |
| Thalamus | 0.026^b^ | 383† | 22/-18/12 | R | 3.79 |
| Ventral striatum |  |  | 14/0/8 | R | 3.49 |
| Inferior temporal gyrus | 0.8833^b^ | 27 | -40/-16/-22 | L | 3.60 |
| Occipital gyrus | 0.7544^b^ | 48 | 26/-68/-4 | R | 3.58 |
| Anterior cingulate cortex | 0.6655^b^ | 62 | -18/34/14 | L | 3.57 |
| Ventral striatum | 0.8543^b^ | 32 | -14/-2/6 | L | 3.56 |
| Inferior frontal gyrus | 0.3252^b^ | 131 | 46/6/14 | R | 3.55 |
| Cerebellum | 0.8239^b^ | 37 | 2/-60/-6 | R | 3.53 |
| Superior temporal gyrus | 0.9104^b^ | 37 | 40/-40/16 | R | 3.52 |
| Lingual gyrus | 0.8661^b^ | 30 | -20/-50/2 | L | 3.44 |
| Insula | 0.8115^b^ | 39 | 28/34/8 | R | 3.31 |

Results are reported using a cluster-forming threshold p<0.001 uncorrected, with an extent threshold of 20 voxels. **^*^**Small volume corrected. ^a^peak-level FWE-corrected, ^b^cluster-level FWE-corrected. † survives FWE correction for multiple comparisons at the cluster or voxel level. No significant effects were found for baseline > follow-up.

**Supplementary Table S3.** *Group effect during adaptive reward prediction at baseline*

| **Healthy controls > Ultra high-risk subjects** | | | | | |
| --- | --- | --- | --- | --- | --- |
| **Region** | **P value** | **Cluster size** | **MNI co-ordinates (X/Y/Z)** | **R/L** | **Z value** |
| Ventral striatum***** | 0.025^a^ | /† | -16/6/-8 | L | 3.54 |
| Parahippocampal gyrus | <0.0001^b^ | 831† | -14/-36/-8 | L | 4.14 |
| Cerebellum |  |  | 2/-58/-10 | L | 3.89 |
| Midbrain |  |  | -8/-30/-8 | L | 3.88 |
| Middle temporal gyrus | 0.042^b^ | 325† | -46/-64/0 | L | 4.03 |
| Superior temporal gyrus | 0.8311^b^ | 36 | -46/14/-14 | L | 3.90 |
| Precentral gyrus | 0.3664^b^ | 119 | -48/-6/40 | L | 3.76 |
| Insula | 0.0735^b^ | 269 | 44/6/-2 | R | 3.64 |
| Ventral striatum | 0.7222^b^ | 53 | -16/6/-8 | L | 3.54 |
| Middle cingulate cortex | 0.6773^b^ | 60 | -6/-12/40 | L | 3.52 |
| Precentral gyrus | 0.8556^b^ | 32 | 46/-8/44 | R | 3.50 |
| Supplementary Motor Cortex | 0.2877^b^ | 141 | 4/6/46 | R | 3.49 |
| Middle cingulate cortex |  |  | 10/10/40 | R | 3.44 |
| Precentral gyrus | 0.7868^b^ | 43 | 54/-2/36 | R | 3.48 |
| Ventral striatum | 0.8904^b^ | 26 | 26/-10/8 | R | 3.46 |
| Insula | 0.5791^b^ | 76 | -40/0/10 | L | 3.45 |
| Calcarine sulcus | 0.8123^b^ | 39 | -12/-80/6 | L | 3.41 |
| Lingual gyrus |  |  | -6/-74/4 | L | 3.16 |
| Ventral striatum | 0.7416^b^ | 50 | -30/-10/-8 | L | 3.31 |

Results are reported using a cluster-forming threshold p<0.001 uncorrected, with an extent threshold of 20 voxels. **^*^**Small volume corrected. ^a^peak-level FWE-corrected, ^b^cluster-level FWE-corrected. † survives FWE correction for multiple comparisons at the cluster or voxel level. No significant effects were found for ultra high-risk subjects > healthy controls.

**Supplementary Table S4.** *Group effect during adaptive reward prediction at follow-up*

| **Healthy controls > Ultra high-risk subjects** | | | | | |
| --- | --- | --- | --- | --- | --- |
| **Region** | **P value** | **Cluster size** | **MNI co-ordinates (X/Y/Z)** | **R/L** | **Z value** |
| Ventral striatum***** | 0.021^a^ | /† | 10/18/-2 | R | 3.60 |
| Ventral striatum***** | 0.044^a^ | /† | -4/14/0 | L | 3.33 |
| Anterior cingulate cortex | 0.0617^b^ | 293 | -16/32/16 | L | 3.93 |
| Inferior frontal gyrus |  |  | -32/20/18 | L | 3.76 |
| Cerebellum | 0.2488^b^ | 157 | -47/-52/-40 | L | 3.89 |
| Ventral striatum | 0.7477^b^ | 49 | 10/18/-2 | R | 3.60 |
| Middle cingulate cortex | 0.8531^b^ | 32 | -18/-40/34 | L | 3.43 |
| Thalamus | 0.3540^b^ | 124 | -4/-12/12 | L | 3.40 |
| Ventral striatum | 0.7856^b^ | 43 | -4/14/0 | L | 3.33 |
| Insula | 0.9141^b^ | 21 | -26/-26/30 | L | 3.30 |
| Cerebellum | 0.8351^b^ | 35 | 38/-48/-38 | R | 3.29 |
| Occipital gyrus | 0.9141^b^ | 21 | 20/-94/10 | R | 3.26 |
| Occipital gyrus | 0.8763^b^ | 28 | -20/-86/14 | L | 3.24 |
| Insula | 0.9192^b^ | 20 | 30/-24/28 | R | 3.21 |

Results are reported using a cluster-forming threshold p<0.001 uncorrected, with an extent threshold of 20 voxels. **^*^**Small volume corrected. ^a^peak-level FWE-corrected. ^b^cluster-level FWE-corrected. † survives FWE correction for multiple comparisons at the voxel level. No significant effects were found for ultra high-risk subjects > healthy controls.

***Supplementary Table S5.*** *Negative correlation between longitudinal changes in activation during adaptive reward prediction and change in the severity of abnormal beliefs in ultra high-risk subjects*

| **Region** | **P value** | **Cluster size** | **MNI co-ordinates (X/Y/Z)** | **R/L** | **Z value** |
| --- | --- | --- | --- | --- | --- |
| Ventral striatum* | 0.0171^a^ | /† | 18/6/-6 | R | 3.73 |
| Precentral gyrus | 0.6126^b^ | 69 | 20/-20/64 | R | 3.79 |
| Ventral striatum | 0.2033^b^ | 160 | 18/6/-6 | R | 3.73 |
| Supplementary Motor Cortex | 0.026^b^ | 340† | 0/-16/58 | R/L | 3.65 |
| Supplementary Motor Cortex |  |  | -10/-4/70 | L | 3.52 |
| Supplementary Motor Cortex |  |  | 4/-6/64 | R | 3.52 |

Results are reported using a cluster-forming threshold p<0.001 uncorrected, with an extent threshold of 20 voxels. **^*^**Small volume corrected. ^a^peak-level FWE-corrected. ^b^cluster-level FWE-corrected. † survives FWE correction for multiple comparisons at the cluster or voxel level. No significant positive correlations were found.

**Supplementary Fig. S1.** Significant correlations in ultra high-risk subjects between explicit adaptive salience responses (visual analogue scale, VAS) and **(A)** unusual thought content (r=-0.674, p<0.001), **(B)** CAARMS positive symptoms (r=-0.653, p<0.001) and **(C)** global functioning (GAF) (r=0.497, p=0.014) at follow-up.

**A)**


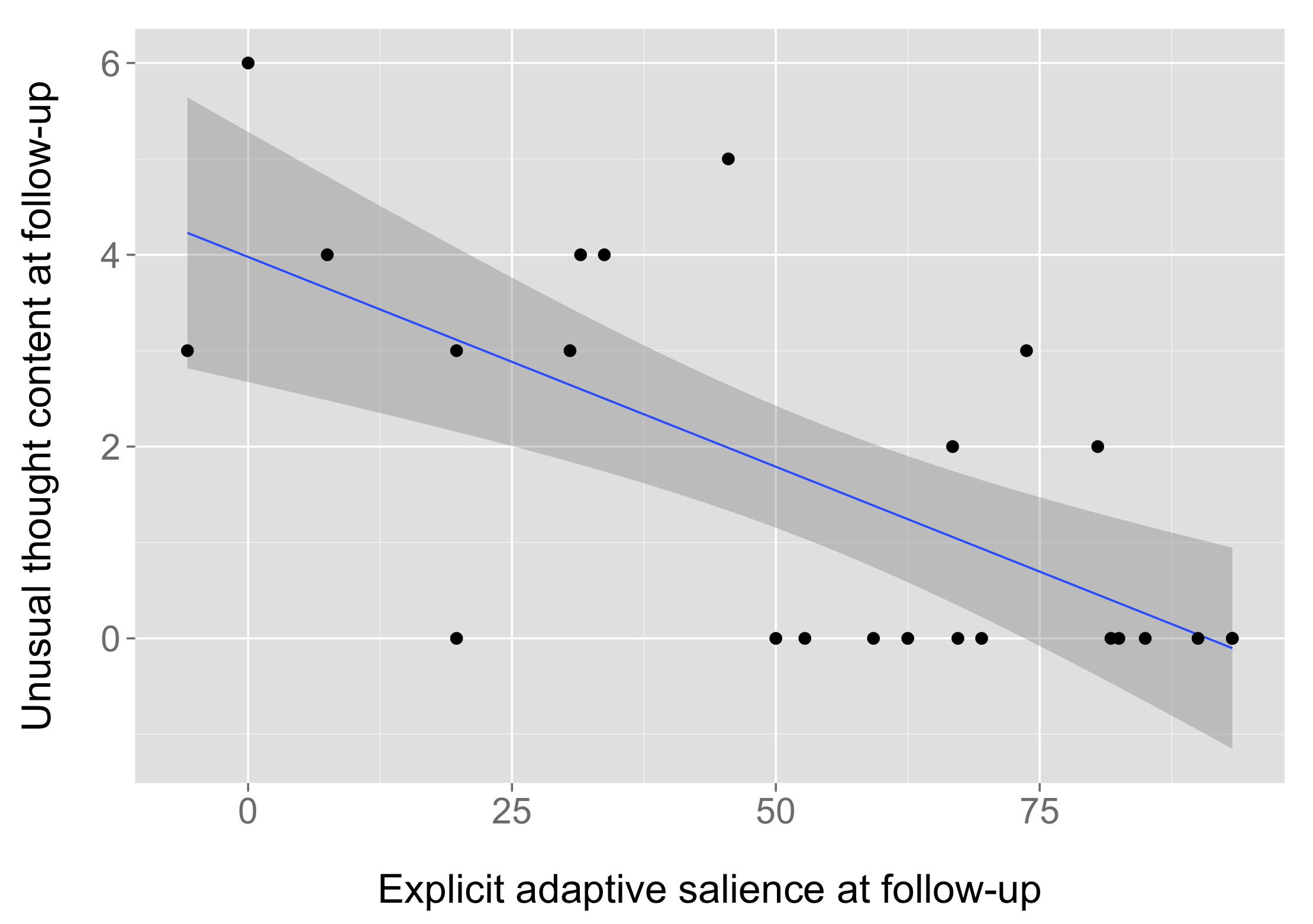


**B)**


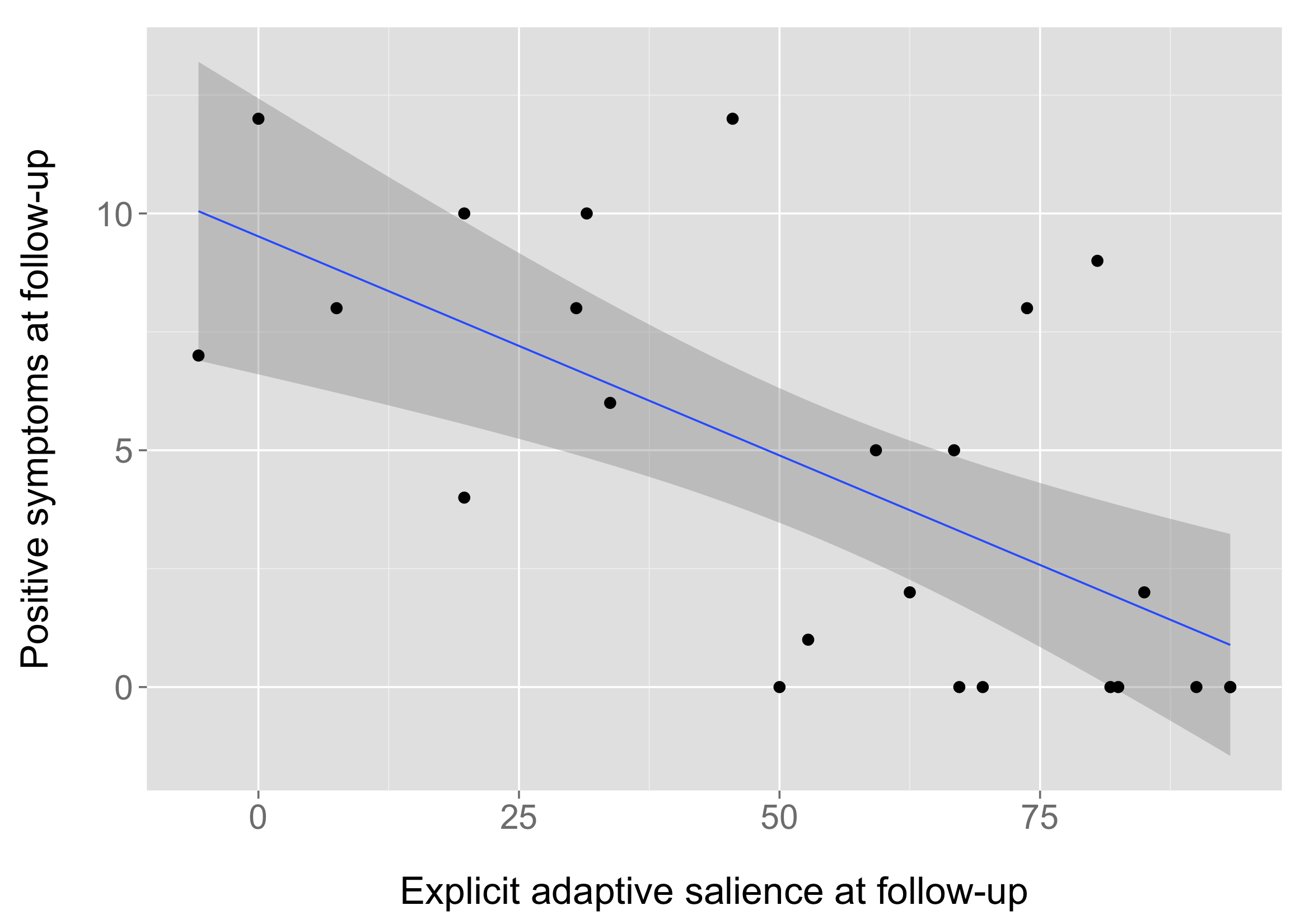


**C)**


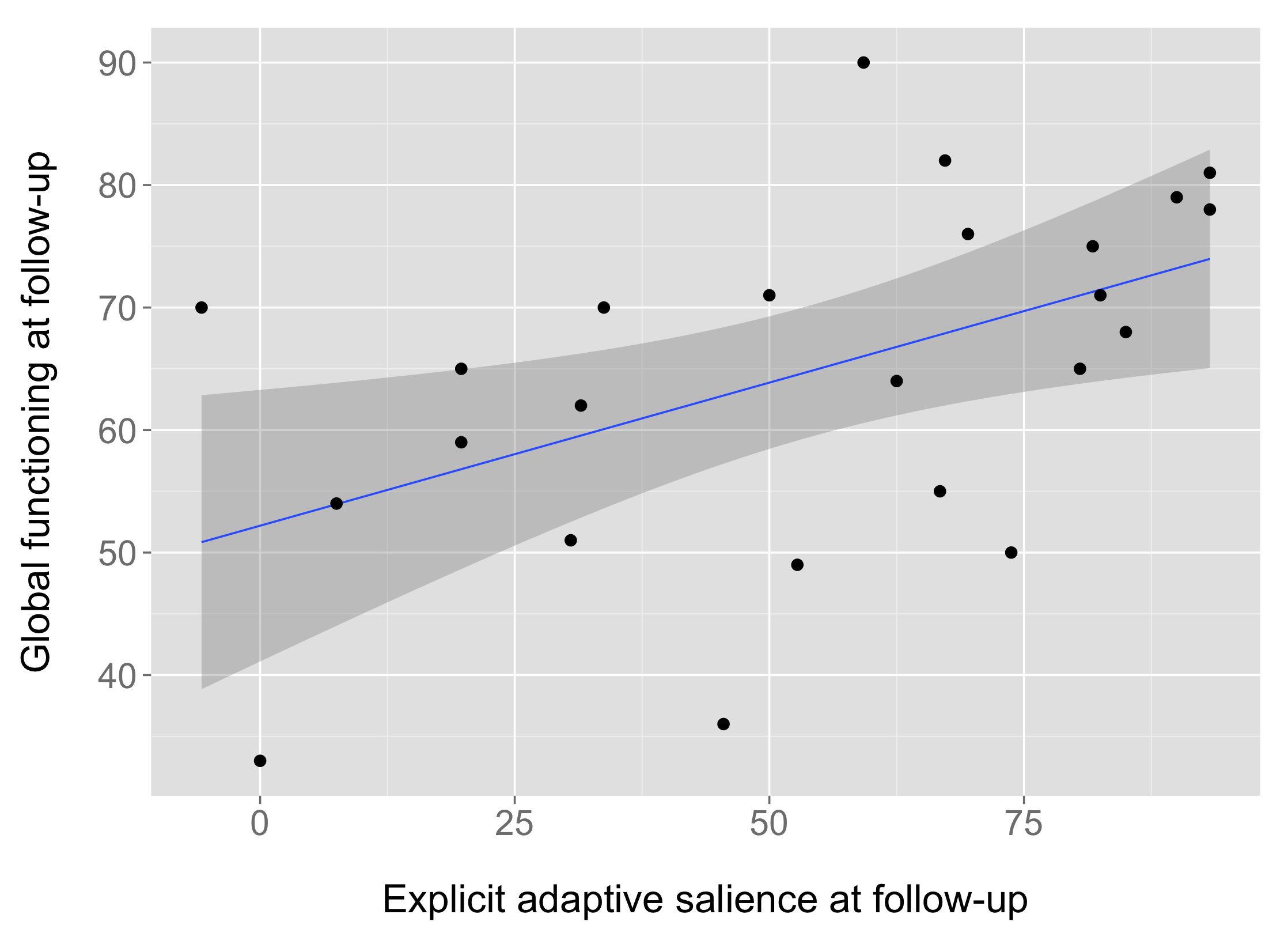


**Supplementary information S2. Results in healthy controls (n=13) and subjects at ultra high-risk for psychosis who did not transit to psychosis (n=22).**

**A. Behavioural data**

*Aberrant attribution of salience*

Across both visits, UHR subjects showed significantly higher implicit aberrant salience than HCs subjects (F(1,33)=6.443, p=0.016), and there was a trend for a group x time interaction (F(1,33)=3.076, p=0.089). There was also a trend for a group x time interaction for explicit aberrant salience (F(1,33)=3.437, p=0.073). Based on our *a priori* hypotheses we constructed linear contrasts at each time point to test for the predicted group differences in aberrant salience.

At baseline, UHR subjects were more likely than HCs to attribute salience to irrelevant cue features (explicit aberrant salience) (F(1,33)=5.117, p=0.030), but did not exhibit greater implicit aberrant salience than HCs (F(1,33)=0.879, p=0.335). At follow-up the group difference in explicit aberrant salience was no longer significant (F(1,33)=0.073, p=0.789), but HCs had significantly lower implicit aberrant scores than the UHR group (F(1,33)=11.972, p=0.002) due to a reduction in this measure over time.

*Adaptive attribution of salience*

Across both visits, the UHR group had lower implicit adaptive salience scores than HCs (F(1,33)=11.603, p=0.002), as well as lower explicit adaptive salience scores (F(1,33)=5.763, p=0.02). There was also a strong trend for a group x time interaction for explicit adaptive salience (F(1,33)=4.086, p=0.051).

At baseline, UHR subjects had significantly lower implicit adaptive salience than HCs (F(1,33)=14.562, p=0.001) and also exhibited significantly lower explicit adaptive salience (F(1,33)=9.391, p=0.004). Both of these group differences were no longer significant at follow-up (implicit adaptive salience: F(1,33)=3.642, p=0.065; explicit adaptive salience: F(1,33)=1.446, p=0.238), due to improved scores in the UHR group together with relatively stable performance in HCs.

**B. Activation during salience processing**

***Supplementary Table S6.*** Group effect across both visits during adaptive reward prediction

| **Healthy controls > Ultra high-risk subjects without transition (n=22)** | | | | | |
| --- | --- | --- | --- | --- | --- |
| **Region** | **P value** | **Cluster size** | **MNI co-ordinates (X/Y/Z)** | **R/L** | **Z value** |
| Ventral striatum***** | 0.025^a^ | /† | 12/2/8 | R | 3.54 |
| Ventral striatum***** | 0.008^a^ | /† | -8/8/4 | L | 3.90 |
| Midbrain | 0.024^b^ | 401† | -6/-24/-10 | L | 4.25 |
| Midbrain |  |  | 10/-24/-12 | R | 3.81 |
| Calcarine sulcus | 0.118^b^ | 231 | 12/-74/14 | R | 4.05 |
| Calcarine sulcus |  |  | 22/-70/6 | R | 3.31 |
| Superior temporal gyrus | 0.619^b^ | 70 | -46/12/-16 | L | 4.02 |
| Insula | 0.514^b^ | 89 | -38/-4/12 | L | 3.97 |
| Ventral striatum | 0.393^b^ | 115 | -8/8/4 | L | 3.90 |
| Cuneus | 0.175^b^ | 192 | -14/-74/24 | L | 3.87 |
| Occipital gyrus |  |  | -26/-76/20 | L | 3.55 |
| Precentral gyrus | 0.253^b^ | 157 | -50/2/36 | L | 3.80 |
|  |  |  | -44/-4/36 | L | 3.59 |
|  |  |  | -46/-6/50 | L | 3.25 |
| Insula | 0.253^b^ | 157 | 44/8/8 | R | 3.80 |
| Inferior frontal gyrus |  |  | 52/10/2 | R | 3.60 |
| Superior patietal gyrus | 0.440^b^ | 104 | -14/-70/44 | L | 3.79 |
| Postcentral gyrus | 0.354^b^ | 125 | -20/-34/54 | L | 3.79 |
| Middle temporal gyrus | 0.070^b^ | 284 | -52/-58/-2 | L | 3.70 |
| Calcarine sulcus | 0.272^b^ | 150 | -8/-78/6 | L | 3.69 |
| Middle cingulate cortex | 0.205^b^ | 177 | 6/6/44 | R | 3.68 |
| Cuneus | 0.350^b^ | 126 | 18/-96/8 | R | 3.60 |
| Occipital gyrus |  |  | 26/-86/6 | R | 3.50 |
| Calcarine sulcus |  |  | 16/-100/0 | R | 3.47 |
| Supramarginal gyrus | 0.846^b^ | 33 | 54/-36/34 | R | 3.55 |
| Ventral striatum | 0.573^b^ | 78 | 12/2/8 | R | 3.54 |
| Inferior parietal gyrus | 0.908^b^ | 22 | -30/-44/36 | L | 3.47 |
| Thalamus | 0.840^b^ | 34 | 12/-14/8 | R | 3.46 |
| Supplementary Motor Cortex | 0.785^b^ | 43 | 2/-16/52 | R | 3.45 |
| Middle cingulate cortex | 0.760^b^ | 47 | -6/-10/40 | L | 3.41 |
| Middle cingulate cortex |  |  | -10/-20/40 | L | 3.25 |
| Ventral striatum | 0.846^b^ | 33 | 12/4/-10 | R | 3.41 |
| Insula | 0.816^b^ | 38 | -30/-22/10 | L | 3.41 |
| Supplementary Motor Cortex | 0.918^b^ | 20 | 18/-22/52 | R | 3.37 |
| Ventral striatum | 0.840^b^ | 34 | -14/8/-8 | L | 3.34 |
| Thalamus | 0.912^b^ | 21 | -4/-20/12 | L | 3.34 |
| Precentral gyrus | 0.816^b^ | 38 | 50/-4/44 | R | 3.33 |
| Inferior frontal gyrus |  |  | 56/8/26 | R | 3.31 |
| Cuneus | 0.869^b^ | 29 | -2/-86/20 | L | 3.32 |

Results are reported using a cluster-forming threshold p<0.001 uncorrected, with an extent threshold of 20 voxels. **^*^**Small volume corrected. ^a^peak-level FWE-corrected, ^b^cluster-level FWE-corrected. † survives FWE correction for multiple comparisons at the cluster or voxel level.

No significant effects were found for ultra high-risk subjects > healthy controls.

***Supplementary Table S7.*** *Time effect across both groups (healthy controls + ultra high-risk subjects without transition) during adaptive reward prediction*

| **Follow-up activity > baseline activity** | | | | | |
| --- | --- | --- | --- | --- | --- |
| **Region** | **P value** | **Cluster size** | **MNI co-ordinates (X/Y/Z)** | **R/L** | **Z value** |
| Ventral part of head of caudate nucleus* | 0.006^a^ | /† | -4/16/-6 | L | 3.99 |
| Ventral part of head of caudate nucleus | 0.037^a^ | /† | 19/12/-2 | R | 3.41 |
| Ventral striatum | 0.125^b^ | 209 | -4/-16/-6 | L | 3.99 |
| Ventral striatum |  |  | -18/24/-8 | L | 3.55 |
| Ventral striatum |  |  | -8/12/-12 | L | 3.43 |
| Inferior frontal gyrus | 0.132^b^ | 211 | 46/6/14 | R | 3.85 |
| Insula |  |  | 38/-16/28 | R | 3.75 |
| Thalamus | 0.0074^b^ | 513† | 22/-18/12 | R | 3.85 |
| Thalamus |  |  | 18/-8/10 | R | 3.69 |
| Ventral striatum |  |  | 14/0/8 | R | 3.62 |
| Insula | 0.411^b^ | 108 | 28/34/8 | R | 3.72 |
| Inferior frontal gyrus |  |  | 38/18/10 | R | 3.19 |
| Lingual gyrus | 0.626^b^ | 68 | 2/-62/-6 | R | 3.71 |
| Occipital gyrus | 0.683^b^ | 59 | 26/-68/-4 | R | 3.65 |
| Cerebellum | 0.868^b^ | 30 | -4/-36/-46 | L | 3.64 |
| Middle frontal gyrus | 0.578 | 76 | -24/34/12 | L | 3.59 |
| Inferior temporal gyrus | 0.902 | 24 | -40/-14/-24 | L | 3.59 |
| Superior temporal gyrus | 0.908^b^ | 23 | 40/-40/16 | R | 3.59 |
| Amygdala | 0.923^b^ | 20 | -16/-6/-10 | L | 3.53 |
| Lingual gyrus | 0.923^b^ | 20 | -20/-50/2 | L | 3.45 |
| Occipital gyrus |  |  | -34/-66/2 | L | 3.27 |
| Ventral striatum | 0.908^b^ | 23 | -14/-2/6 | L | 3.42 |
| Ventral striatum | 0.880^b^ | 28 | 30/12/6 | R | 3.24 |
|  |  |  | 24/18/6 | R | 3.15 |

Results are reported using a cluster-forming threshold p<0.001 uncorrected, with an extent threshold of 20 voxels. **^*^**Small volume corrected. ^a^peak-level FWE-corrected, ^b^cluster-level FWE-corrected. † survives FWE correction for multiple comparisons at the cluster or voxel level. No significant effects were found for baseline > follow-up.

***Supplementary Table S8.*** *Group effect during adaptive reward prediction at baseline*

| **Healthy controls > Ultra high-risk subjects without conversion (n=22)** | | | | | |
| --- | --- | --- | --- | --- | --- |
| **Region** | **P value** | **Cluster size** | **MNI co-ordinates (X/Y/Z)** | **R/L** | **Z value** |
| Ventral striatum***** | 0.009^a^ | /† | -16/6/-10 | L | 3.87 |
| Ventral striatum***** | 0.042^a^ | /† | 14/2/8 | R | 3.37 |
| Precentral gyrus | 0.069^b^ | 268 | -50/0/38 | L | 4.37 |
| Precentral gyrus |  |  | -42/-10/44 | L | 3.41 |
| Postcentral gyrus |  |  | -44/-20/46 | L | 3.29 |
| Parahippocampal gyrus | <0.0001^b^ | 1117† | -12/-36/-8 | L | 4.32 |
| Cerebellum |  |  | 0/-40/-12 | L | 4.16 |
| Midbrain |  |  | 8/-30/-10 | R | 3.83 |
| Superior temporal gyrus | 0.014^b^ | 428† | -46/14/-14 | L | 4.25 |
| Ventral striatum |  |  | -16/6/-10 | L | 3.87 |
| Ventral striatum |  |  | -24/-2/-10 | L | 3.81 |
| Insula | 0.106^b^ | 228 | -38/-2/12 | L | 4.17 |
| Inferior frontal gyrus |  |  | -52/6/10 | L | 3.50 |
| Middle temporal gyrus | 0.052^b^ | 295 | -46/-64/0 | L | 4.08 |
| Middle temporal gyrus |  |  | -44/-44/-2 | L | 3.37 |
| Middle temporal gyrus |  |  | -50/-50/-4 | L | 3.24 |
| Precentral gyrus | 0.243^b^ | 153 | 46/-8/44 | R | 4.00 |
| Precentral gyrus |  |  | 54/2/36 | R | 3.62 |
| Precentral gyrus |  |  | 56/8/30 | R | 3.51 |
| Middle cingulate cortex | 0.036^b^ | 332† | 8/10/40 | R | 3.82 |
| Ventral striatum | 0.473^b^ | 94 | 26/-10/8 | R | 3.82 |
| Ventral striatum |  |  | 22/-2/8 | R | 3.52 |
| Ventral striatum |  |  | 12/4/6 | R | 3.32 |
| Middle cingulate cortex | 0.270^b^ | 144 | -6/-12/40 | L | 3.74 |
| Insula | 0.048^b^ | 304† | 44/6/-2 | R | 3.62 |
| Insula |  |  | 42/8/6 | R | 3.61 |
| Inferior frontal gyrus |  |  | 56/6/8 | R | 3.55 |
| Precentral gyrus | 0.893^b^ | 26 | -22/-28/52 | L | 3.43 |
| Calcarine sulcus | 0.876^b^ | 29 | -12/-80/6 | L | 3.38 |
| Insula | 0.910^b^ | 23 | -32/-22/10 | L | 3.34 |

Results are reported using a cluster-forming threshold p<0.001 uncorrected, with an extent threshold of 20 voxels. **^*^**Small volume corrected. ^a^peak-level FWE-corrected, ^b^cluster-level FWE-corrected. † survives FWE correction for multiple comparisons at the cluster or voxel level. No significant effects were found for ultra high-risk subjects > healthy controls.

**Supplementary Table S9.** *Group effect during adaptive reward prediction at follow-up*

| **Healthy controls > Ultra high-risk subjects without conversion (n=22)** | | | | | |
| --- | --- | --- | --- | --- | --- |
| **Region** | **P value** | **Cluster size** | **MNI co-ordinates (X/Y/Z)** | **R/L** | **Z value** |
| Ventral striatum***** | 0.025^a^ | /† | 10/18/-2 | R | 3.53 |
| Ventral striatum***** | 0.046^a^ | /† | -6/14/0 | L | 3.31 |
| Anterior cingulate cortex | 0.089^b^ | 255 | -16/32/16 | L | 3.86 |
| Inferior frontal gyrus |  |  | -32/20/18 | L | 3.72 |
| Ventral striatum |  |  | -20/14/20 | L | 3.42 |
| Cerebellum | 0.264^b^ | 151 | -46/-52/-40 | L | 3.82 |
| Ventral striatum | 0.811^b^ | 39 | 10/18/-2 | R | 3.53 |
| Middle cingulate cortex | 0.914^b^ | 21 | -18/-40/34 | L | 3.38 |
| Thalamus | 0.446^b^ | 102 | -4/-12/12 | L | 3.35 |
| Thalamus |  |  | -12/-4/10 | L | 3.32 |
| Ventral striatum | 0.754^b^ | 48 | -6/14/0 | L | 3.31 |
| Insula | 0.920^b^ | 20 | -26/-26/30 | L | 3.31 |
| Cerebellum | 0.899^b^ | 24 | 38/-48/-38 | R | 3.24 |

Results are reported using a cluster-forming threshold p<0.001 uncorrected, with an extent threshold of 20 voxels. **^*^**Small volume corrected. ^a^peak-level FWE-corrected. ^b^cluster-level FWE-corrected. † survives FWE correction for multiple comparisons at the voxel level. No significant effects were found for ultra high-risk subjects > healthy controls.

**Supplementary Table S10.** *Negative correlation between longitudinal changes in activation during adaptive reward prediction and change in the severity of abnormal beliefs in ultra high-risk subjects who did not convert (n=22)*

| **Region** | **P value** | **Cluster size** | **MNI co-ordinates (X/Y/Z)** | **R/L** | **Z value** |
| --- | --- | --- | --- | --- | --- |
| Ventral striatum* | 0.021^a^ | /† | 18/6/-6 | R | 3.67 |
| Supplementary Motor Cortex | 0.001^b^ | 671† | 4/-6/62 | R | 4.18 |
| Supplementary Motor Cortex |  |  | -8/-6/70 | L | 4.13 |
| Supplementary Motor Cortex |  |  | -2/-14/58 | L | 3.41 |
| Ventral striatum | 0.174^b^ | 171 | 32/0/2 | R | 3.75 |
| Ventral striatum |  |  | 18/6/-6 | R | 3.67 |
| Ventral striatum | 0.745^b^ | 50 | -30/-20/0 | L | 3.30 |
| Ventral striatum |  |  | -26/-8/-4 | L | 3.18 |

Results are reported using a cluster-forming threshold p<0.001 uncorrected, with an extent threshold of 20 voxels. **^*^**Small volume corrected. ^a^peak-level FWE-corrected. ^b^cluster-level FWE-corrected. † survives FWE correction for multiple comparisons at the cluster or voxel level. No significant positive correlations were found.
